# c‐Src activation as a potential marker of chemical‐induced skin irritation using tissue‐engineered skin equivalents

**DOI:** 10.1111/exd.14719

**Published:** 2022-12-11

**Authors:** Amy L. Harding, Helen E. Colley, Inmaculada Barragan Vazquez, Simon Danby, Md Zobaer Hasan, Hirofumi Nakanishi, Tetsuo Furuno, Craig Murdoch

**Affiliations:** ^1^ School of Clinical Dentistry University of Sheffield Sheffield UK; ^2^ Sheffield Dermatology Research, Department of Infection, Immunity & Cardiovascular Disease, Medical School University of Sheffield Sheffield UK; ^3^ Rohto Pharmaceutical Co., Ltd., Safety Design Centre Kyoto Japan

**Keywords:** c‐Src, irritation, signal transduction, skin, toxicity

## Abstract

Skin irritancy to topically applied chemicals is a significant problem that affects millions of people worldwide. New or modified chemical entities must be tested for potential skin irritancy by industry as part of the safety and toxicity profiling process. Many of these tests have now moved to a non‐animal‐based format to reduce experiments on animals. However, these tests for irritancy potential often rely on monolayer cultures of keratinocytes that are not representative of the skin architecture or tissue‐engineered human skin equivalents (HSE) using complex multi‐gene expression panels that are often cumbersome and not amenable for high throughput. Here, we show that human skin equivalents increase abundance of several phosphorylated kinases (c‐Src, c‐Jun, p53, GSK3α/β) in response to irritant chemical stimulation by phosphokinase array analysis. Specific phosphorylation of c‐Src^Y419^ was confirmed by immunoblotting and was plasma membrane‐associated in basal/spinous cells by phospho‐specific immunohistochemistry. Moreover, c‐Src^Y419^ phosphorylation in response to the irritants lactic acid and capsaicin was inhibited by the c‐Src inhibitors KB‐SRC and betaine trimethylglycine. These data provide the first evidence for c‐Src specific activation in response to chemical irritants and point to the development of new modes of rapid testing by immunodetection for first‐pass screening of potential irritants.

## Abstract 2

This study demonstrates that specific kinases, in particular c‐Src, are activated in HSE in response to chemical irritants. The rapid increase in specific phosphorylated signal transduction molecules upon exposure to irritants may prove ideal targets to rapidly identify and differentiate potential chemical irritants from non‐irritants.

## 1 BACKGROUND

Chemical‐induced skin irritancy or sensitivity to topically applied dermatological agents, cosmetics, skin‐care products, detergents or other compounds is a common problem. Skin sensitivity relates only to those individuals who are susceptible to a particular chemical, whereupon it produces paresthetic sensations, whereas skin irritation is related to the characteristics of a chemical that, when applied, will induce irritation on all subjects.^1^ Several patient or animal‐based assays have been used to identify potentially irritant chemicals, but none of these tests provide a clear standardised measurable outcome and are not compatible with high‐throughput testing that is required by industry, particularly for first‐pass drug screening. This, along with the legislative move toward non‐animal testing, has prompted the development of several in vitro assays for skin irritation.

Examples based on monolayer keratinocyte luciferase reporter‐based assay systems include KeratinoSens™^2^, ^3^ and LuSens,^4^ where chemically induced activation of the transcription factor, nuclear factor erythroid 2 (Nrf2) mediates its translocation to the nucleus, where it heterodimerises with other transcription factors (Mif/c‐Jun) and binds to the antioxidant response element (ARE) in the promoter region, driving reporter luciferase expression. However, these tests are based on monolayer culture, whereas skin is comprised of a stratified squamous epithelium containing keratinocytes which display increasing levels of differentiation and keratinisation that play a major role in skin permeability to topically applied chemicals. The deficiencies of simple monoculture assays can be overcome by the use of tissue‐engineered human skin equivalents (HSE) that accurately mimic the structure of human skin for skin irritancy testing.

A number of studies have used HSE to identify increased expression of several genes in response to many chemicals and known irritants.^5^, ^6^, ^7^, ^8^ However, these gene signature sets are often large comprising of between 7 and 25 genes that would be cumbersome for high‐throughput screening. Analysing the immediate up‐stream signalling cascade events upon treatment with a chemical irritant may not only identify activation of specific kinases in the epidermis that are important in irritant‐response pathways but may also ascertain markers of irritancy that could be tested in more rapid assay formats.

## 2 QUESTION ADDRESSED

Do chemicals that cause skin irritancy induce common intracellular signalling events such as protein kinase phosphorylation and can these cellular events be used as biomarkers for chemicals with skin irritancy potential?

## 3 EXPERIMENTAL DESIGN

See Supporting Information.

## 4 RESULTS

**FIGURE 1:**
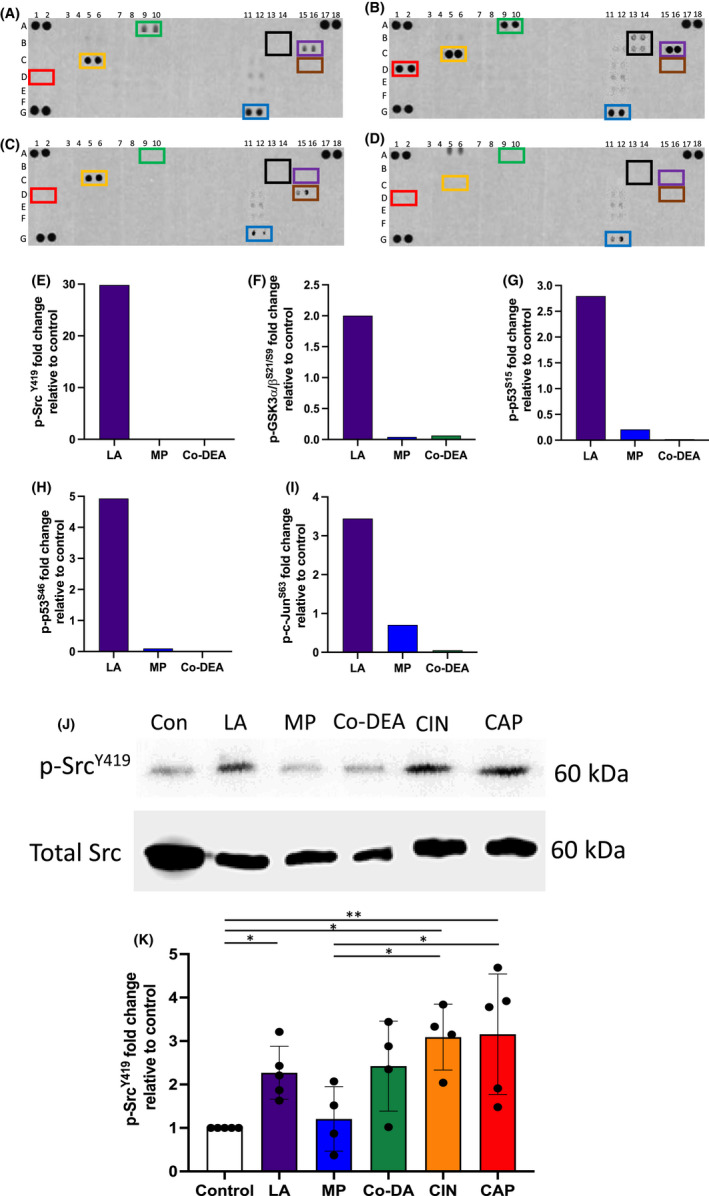
Phosphokinase array analysis of human skin equivalents (HSE). Representative array immunoblots showing levels of phosphorylated kinases in the total protein extracts of HSE following 15 minutes treatment with H_2_O as control (A), LA (B), MP (C) or Co‐DEA (D). Colour code for highlighted boxes is: 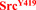, 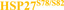, 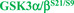, 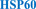, 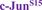, 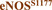. Densitometry analysis of array immunoblots showing fold‐change abundance of phosphokinases in LA, MP or CO‐DEA treated HSE relative to water controls for Src^Y419^ (E), GSK3α/β^S21/S9^ (F), p53^S15^ (G), p53^S46^ (H) and c‐Jun^S63^ (I) (*n* = 2 independent experiments). Immuno‐blot analysis of HSE for phospho‐c‐Src^Y419^ abundance compared with total c‐Src upon stimulation with water control (con), LA, MP, Co‐DEA, CIN or CAP for 15 min (J). Densitometric analysis showing fold change in phospho‐c‐Src^Y^ ^419^ relative to total c‐Src (K). Data are mean ± SD for *n* = 3 independent experiments *p < 0.05, **p < 0.01 by one‐way ANOVA with Tukey's multiple comparison post hoc test. The map accompanying the phosphokinase array is shown in Figure S2.

Phosphokinase array analysis of HSE protein extracts showed a dramatic and significant increased abundance of phospho‐c‐Src^Y419^ when the known skin irritant, lactic acid (LA), was topically applied to HSE for 15 minutes, in comparison to the non‐irritants methylparaben (MP) and cocamide diethanolamine (Co‐DEA), or water applied as carrier control (Figure 1). In our previous study, we identified a seven‐gene signature panel to identify chemical irritant from non‐irritant.^8^ Four of the genes identified in this panel (*IL‐6, PTGS2, MAP3K8, MMP‐3*) are regulated by activation of the transcription factors AP‐1 (c‐Fos/c‐Jun) and p65/NFκB. In line with these data, we found increased phosphorylation of both c‐Jun^S63^ (Figure 1) and p65^S536^ (Figure S1) in response to irritants but not non‐irritants. In addition to phospho‐c‐Src^Y419^, increased abundance of other phosphorylated kinases including phospho‐glycogen synthase kinase‐3 (GSK3)‐α/β and phospho‐p53 were also markedly increased in the array compared with both non‐irritants and water control (Figure 1). Phosphorylated heat shock proteins 27 and 60 were abundant in the control, LA and MP but not Co‐DEA (Figure 1). The majority of kinases displayed no difference in phosphorylation status between treatments.

Phospho‐specific immunoblot analysis for c‐Src^Y419^ confirmed the array data, showing significantly increased abundance of phospho‐c‐Src^Y419^ for the irritant LA, and even more so in response to cinnamaldehyde (CIN) and capsaicin (CA) stimulation, when compared with MP, Co‐DEA and water control. However, this difference was not as apparent as observed in the array, with the non‐irritant MP displaying levels of phospho‐c‐Src^Y419^ similar to the control and Co‐DEA displaying similar levels to LA (Figure 1J,K). This is likely due to differences in the binding affinity of the two anti‐phospho‐c‐Src specific antibodies used in the two immunoblot methods.

To our knowledge, this is the first observation of c‐Src‐induced phosphorylation in response to irritants by HSE. Moreover, phosphorylation was at tyrosine 419, the main site of phosphorylation within the activation loop that results in Src autophosphorylation and activation status.^9^ c‐Src is a ubiquitously expressed non‐receptor protein tyrosine kinase that is phosphorylated and activated by other protein kinases (e.g., activated epidermal growth factor receptor, adhesion and cytokine receptors as well as several G‐protein‐coupled receptors). Upon activation c‐Src acts by phosphorylating other proteins involved in regulating cell morphology, adhesion, motility, apoptosis, proliferation and survival.^10^ There is currently no evidence for a role of c‐Src in skin irritancy, although this signalling molecule does appear to be important in hyperproliferative epidermal disorders and epidermal wound healing.^11^, ^12^

**FIGURE 2:**
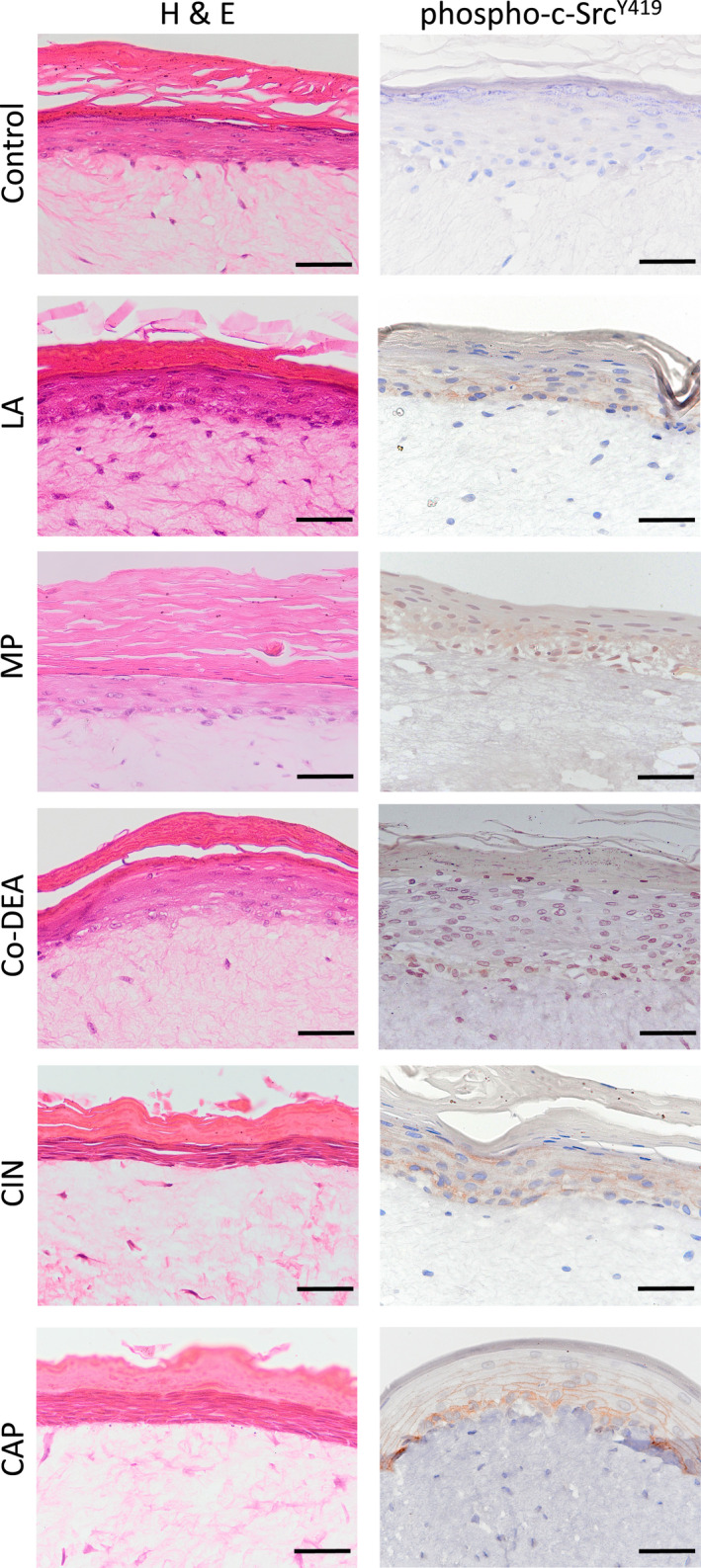
Abundance of phospho‐c‐Src^Y419^ in tissue‐engineered human skin equivalents (HSE) in response to irritants. HSE were generated by culturing N/TERT‐1 immortalised keratinocytes on top of a human dermal fibroblast‐populated collagen scaffold before treatment with water control, LA, MP, Co‐DEA, CIN, or CAP for 15 min. Histological (haematoxylin and eosin, H&E) and immunohistochemical analysis for phospho‐c‐Src^Y419^. Representative images are from three independent experiments. Scale bar = 50 μm.

Histological analysis of HSE sections showed no difference in skin structure between the treatments (Figure 2). Consistent with the immunoblotting data, immunohistochemical staining of tissue sections for phospho‐c‐Src^Y419^ showed that the activated kinase was present in the cytoplasm of basal cells and predominant in the membrane‐associated regions of supra‐basal keratinocytes. Weak and sporadic phospho‐c‐Src‐positive staining was observed in the nuclear and cytoplasmic regions of basal and supra‐basal keratinocytes in MP and Co‐DEA‐treated HSE while no staining was observed in the water control treated HSE (Figure 2). Previous studies have shown activated c‐Src localisation in the nucleus, where it is proposed to play a role in several cellular processes such as regulation of gene transcription, interaction with other nuclear proteins or in mechanotrasnduction, although experiments on nuclear‐located c‐Src have mainly been performed on cancer cells.^13^

The presence of membrane‐associated phospho‐c‐Src^Y419^ in irritant‐treated HSE suggests that c‐Src is recruited to up‐stream membrane‐bound kinases where it is phosphorylated as part of the irritant response process. Activated phospho‐c‐Src^Y419^ is then likely to mediate its actions by phosphorylating down‐stream signalling proteins involved in a number of key pathways in skin keratinocytes. For example, phospho‐c‐Src is known to target cell membrane‐associated signalling factors such as β‐catenin for phosphorylation at cell–cell adheren junctions. Phosphorylation of β‐catenin by phospho‐c‐Src reduces its ability to associate with membrane‐bound E‐cadherin, thereby adversely affecting cell–cell adhesion and epithelial integrity.^14^ Increased abundance of cytoplasmic β‐catenin available for Wnt signalling might be increased further by the elevated levels of phospho‐GSK3α/β^S21/S9^ that were also observed in irritant‐stimulated skin models. This is because phosphorylation at serine 9 and 21 inhibits GSK3α/β activity, preventing β‐catenin degradation via the ubiquitin/proteasome pathway and allowing β‐catenin to enter the nucleus to alter gene transcription.^15^ Src activation also phosphorylates the gap junction protein, connexin 43, triggering gap junction closure that prevents cell to cell transfer of material, thereby attenuating damage to surrounding cells^16^, ^17^; a process which may also occur in chemically challenged keratinocytes. Further experiments examining the consequences of irritant‐stimulated phospho‐c‐Src activation in terms of downstream cell signalling events is warranted. It will be also important to determine if the weak and sporadic nuclear localisation of phospho‐c‐Src upon stimulation with MP and Co‐DEA is a common feature to other non‐irritant chemical entities or is a non‐specific phenomenon.

**FIGURE 3:**
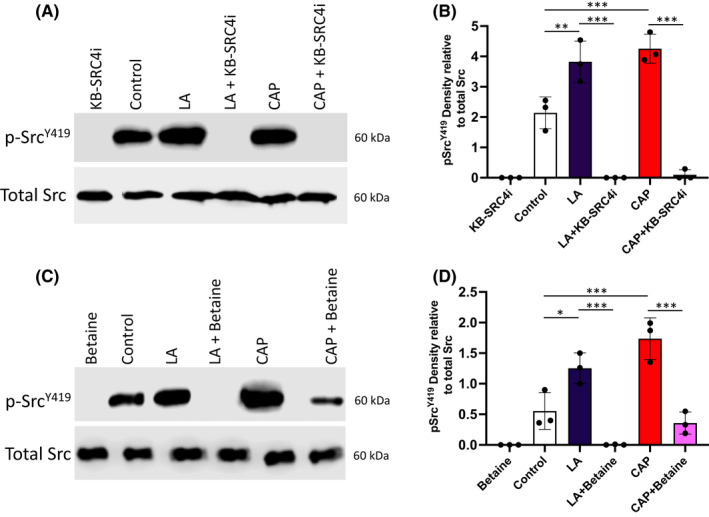
c‐Src inhibitors prevent its phosphorylation in response to irritants. Immunoblots of phospho‐c‐Src^Y419^ abundance compared with total c‐Src upon stimulation with water control, LA, or CAP for 15 min in the absence or presence (1‐h preincubation) of the selective c‐Src inhibitor KB‐SRC4i (A) with accompanying densitometric analysis (B) or with betaine trimethylglycine (C) with accompanying densitometric analysis (D). Representative images are from three independent experiments. Densitometry analysis for both KB‐SRC4i and betaine trimethylglycine is displayed as phospho‐c‐Src^Y419^ band density relative to total c‐Src. Data are mean ± SD for *n* = 3 independent experiments *p < 0.05, **p < 0.01 and ***p < 0.001 by one‐way ANOVA with Tukey's multiple comparison post hoc test.

KB‐SRC is a potent and selective inhibitor of c‐Src phosphorylation and activation with a K_i_ of 44 nM.^18^ Pretreatment of HSE for 1 hour with KB‐SRC completely abrogated the phosphorylation of c‐Src^Y419^ in response to 15‐min stimulation with the irritants LA and CAP (Figure 3A,B), confirming the specificity of the c‐Src^Y419^ phosphorylation response. Interestingly, similar inhibition data was observed when HSE were preincubated with the methyl donor betaine trimethylglycine, a molecule known for its role in DNA methylation, oxidative stress and inhibition of the NF‐κB and NLRP3 pathway^19^ (Figure 3C,D). Incubation with betaine trimethylglycine markedly reduced irritant‐induced phosphorylation of c‐Src^Y419^ in a similar manner to that found previously in hepatocytes.^20^

The activity of c‐Src is increased in response to several genotoxic chemicals and oxidative stress molecules where it links with down‐stream activation of mitogen‐activated protein kinases (MAPKs).^21^ Indeed, constitutive activation of c‐Src is linked to tumorigenesis in skin cancer where it is highly expressed.^22^ Moreover, activated c‐Src is involved in the regulation of *PTGS2* expression,^23^ linking it to the up‐regulation of the genes identified in our seven gene signature panel for irritant potential.^8^ It is possible the rapid phosphorylation of c‐Src by skin irritants is a central step in response to such substances. If so, this opens up potential therapeutic or preventative intervention by topical application of c‐Src inhibitors.

The advantage of searching for up‐stream phosphorylated signal transduction targets is that activation is quick – within minutes; additionally, the activity is largely restricted to the epidermis so the cell lysate can be subjected to rapid testing formats such as immuno‐based rapid antigen tests, rather than rely of complex multi‐gene panel analysis. Further screening against a large panel of irritant/non‐irritant chemicals is now required to verify these findings.

## 5 CONCLUSIONS AND PERSPECTIVES

This study demonstrates that specific kinases, in particular c‐Src, are activated in HSE in response to chemical irritants. c‐Src has been implicated in maintaining skin health, in particular wound healing and keratinocyte proliferation, but its actions in response to chemical insult is unknown. The rapid increase in specific phosphorylated signal transduction molecules or transcription factors upon exposure to irritants may prove ideal targets to rapidly identify and differentiate potential chemical irritants from non‐irritants. They may also lend themselves to more rapid analytical techniques such as antibody‐mediated lateral flow tests.

## AUTHOR CONTRIBUTIONS

ALH, IBV performed the experiments and, along with CM and HEC, analysed the data. CM, HEC, SD, MZH, HN, TF designed the research study. HEC and CM supervised the research. ALH, HEC and CM drafted the manuscript. All researchers edited the final version of the manuscript.

## ACKNOWLEDGMENTS

No further acknowledgments are required.

## FUNDING INFORMATION

This study was funded by Rohto Pharmaceutical Company Limited in conjunction with The University of Sheffield and an Engineering and Physical Sciences Research Council (EPSRC) Impact Acceleration Account award.

## CONFLICT OF INTEREST

MZH, HN, and TF are employees of Rohto Pharmaceutical Company Limited, the industrial partner for the project. The remaining authors state no conflict of interest.

## Supporting information


**Appendix S1.** Experimental design.

**Figure S1.** Chemical skin irritants induce phosphorylation of p65 NFκB.
**Figure S2.** Phosphokinase array map showing target/control coordinates and antibody‐specific phosphorylation sites.

## Data Availability

The data that support the findings of this study are available from the corresponding author upon reasonable request.
