# Supplementary material for: c‐Src activation as a potential marker of chemical‐induced skin irritation using tissue‐engineered skin equivalents
Source: Exp Dermatol. 2022 Dec 11;32(2):220–5. doi: 10.1111/exd.14719 (PMC10946902; doi:10.1111/exd.14719)
Supplement: Supplementary file 1 — Appendix S1. Experimental design. [file EXD-32-220-s001.docx]

**e-Supporting Information.**

**MATERIALS AND METHODS**

All reagents were purchased from Sigma-Aldrich (Dorset, UK) and used to the manufactures’ instructions, unless stated otherwise.

*Cell culture*

TERT-immortalized human skin keratinocytes (KC) (N/TERT-1; from Prof. Rheinwald)^1^ were cultured at low density in KC serum-free media (ThermoFisher Scientific, Waltham, MA) supplemented with 25 mg/ml bovine pituitary extract, 0.2 ng/ml EGF, and 0.3 mM calcium chloride (total calcium ion concentration, 0.4 mM). Human dermal fibroblasts (HDF) were isolated from skin biopsies obtained from the breast tissue of patients undergoing surgery with written, informed consent (ethical approval 09/H1308/66). Biopsies were incubated in 0.1% (w/v) trypsin solution supplemented with 100 IU/ml penicillin, 100 mg/ml streptomycin, and 0.625 mg/ml amphotericin B overnight at 4 ^O^C. After enzymatic digestion, HDF were isolated from the connective tissue by fine mincing followed by treatment with 0.25% (w/v) collagenase for 5 hours at 37 ^O^C then collected by centrifugation and cultured in DMEM supplemented with 10% v/v foetal bovine serum, 100 IU/ml penicillin, 100 mg/ml streptomycin, and 2 mM L-glutamine. Cells were incubated at 37°C in a 5% carbon dioxide humidified incubator, medium changed every 3 days.

*Generation of tissue-engineered human skin equivalents*

Human skin equivalents (HSE) were constructed using techniques previously described.^1,2^ In brief, rat-tail collagen (5 mg/ml) was combined with 8% (v/v) foetal bovine serum, DMEM (10x), 2 mM L-glutamine, and reconstitution buffer (2.2% sodium bicarbonate, 4.8% HEPES, 0.25% sodium hydroxide in distilled H_2_O) and the pH adjusted to 7.4 with 2 M NaOH. Human dermal fibroblasts (HDF; 1.5 x 10^5^ per model) were added to the collagen mixture before transferring into 12-well translucent transwell inserts with 0.4 mm pores (Millipore, Burlington, MA) and allowed to set in a humidified atmosphere at 37°C. Once set, 5 mL keratinocyte culture media was added to the well and 500 μL onto the surface of the collagen and incubated for 24 hours at 37°C. Next, 2.5 x10^5^ N/TERT-1 cells were seeded on the collagen surface and cultured submerged in medium for 2 days, after which HSEs were raised to an air-to-liquid interface and further cultured for 14 days, changing the medium every other day.

*HSE stimulation with chemical compounds*

Fifty μL of chemical compound was added to the surface of HSE and incubated for 15 minutes at 37°C. Chemicals tested were lactic acid (LA; 5% v/v), methylparaben (MP; 0.2% w/v), cocamide diethanolamine (Co-DEA; 2% v/v), capsaicin (CAP; 0.1% w/v), and cinnamaldehyde (CA; 3% v/v). Chemicals were made up in H_2_O, which was also used as a vehicle control (Con). For the c-Src inhibition experiments, HSE were treated topically with 100 μM KB-SRC 4 (Tocris, UK) or 8.53 mM betaine trimethylglycine (Sigma, UK) for 1 hour before addition of lactic acid (LA; 5% v/v), capsaicin (CAP; 0.1% w/v) or water as control.

*Phosphokinase array screen*

Total protein lysates from HSE were prepared in cold radioimmunoprecipitation assay (RIPA) buffer supplemented with Halt™ protease and phosphatase inhibitor cocktail (ThermoFisher Scientific, Waltham, MA, USA) and protein content determined by bicinchoninic acid assay. A human phospho-kinase array profiler (R&D systems), capable of detecting phosphorylation of 37 human kinases was performed according to the manufacturer’s instructions (Figure S2). Nitrocellulose membranes spotted with phospho-kinase specific antibodies were incubated with blocking buffer for 1 hour at room temperature. Tissue lysates (400 μg protein) were mixed with a cocktail of biotinylated detection antibodies, added to the array membrane and incubated overnight at 4°C. Membranes were washed three times in buffer then incubated with streptavidin-conjugated horseradish peroxidase followed by chemiluminescent detection reagent and membranes exposed to x-ray film for development. The relative abundance of each phospho-kinase as determined by densitometry using QuantityOne software (Biorad, USA).

*Immunoblotting*

Protein lysates (40 μg) were resolved by NuPAGE™ 3–8% (w/v) Tris-Acetate 1.0 mm gel (Invitrogen™, ThermoFisher Scientific, Waltham, MA USA) electrophoresis (SDS-PAGE) in NuPAGE™ Tris-Acetate SDS Running Buffer (20X) (Invitrogen™, ThermoFisher Scientific, Waltham, MA USA). Proteins were transferred onto a nitrocellulose membrane (0.2 µm) using a trans-blot turbo mini transfer system (Bio-Rad, Hercules, California, USA) and incubated in blocking solution (5% (w/v) milk powder in Tris-buffered saline (TBS) with 0.05% (v/v) Tween-20 (TBS-T) for 1 hour. Primary rabbit monoclonal antibodies directed against phosphorylated c-Src^Y419^ (Cell Signalling clone D49G4) or phospho-NF-κB^S536^ (Cell Signalling, clone 93H1) were diluted 1 in 1000 in blocking solution and incubated with membranes at 4°C overnight. The membrane was washed 3 × 10 minutes with TBS-T and subsequently incubated with a horseradish peroxidase-conjugated secondary antibody (1:3000 in blocking solution), for 1 hour at room temperature. The membrane was washed 3 × 10 minutes in TBS-T and developed using enhanced chemiluminescence (ECL; Pierce™, ThermoFisher Scientific, Waltham, MA USA). Nitrocellulose membranes were stripped by incubation in Re-Blot Plus Western blot strong antibody stripping solution (MerckMillipore, Burlington, MS, USA) at room temperature for 10 minutes, before being washed, blocked and re-probed with either total c-Src (Cell Signalling, clone 2123) or total NF-κB (Cell Signalling, clone D14E12) diluted 1 in 1000 in blocking solution. The relative abundance of each phosphokinase compared to total kinase was determined by densitometry using QuantityOne software (Biorad, USA).

*Histological analysis*

HSE were fixed with 10% v/v neutral-buffered formalin; alcohol-processed, paraffin wax embedded, 6 µm sections cut using a microtome, and sections stained with haematoxylin and eosin. Microscope slides were mounted with distyrene-polystrene xylene (DPX) and imaged by light microscopy (Olympus BX51 microscope and Colourview IIIu camera with associated Cell^D software) (Olympus soft imaging solutions, GmbH, Germany).

*Immunohistochemistry*

Paraffin-embedded tissue sections (6 μm) were dewaxed, rehydrated and endogenous peroxidase neutralised with 3% hydrogen peroxide for 20 minutes. Antigen retrieval was performed using sodium citrate buffer (10 mM sodium citrate, 0.05% Tween, pH 6.0). Sections were blocked using protein-free blocking solution (Dako, Agilent, California, USA) for 30 minutes at room temperature before incubation with primary antibody, recombinant anti-phospho-c-Src^Y419^ antibody, (Abcam, clone EPR17734) for 1 hour at room temperature. Secondary antibody and avidin-biotin complex (ABC) provided with Vectastain Elite ABC kit (Vector laboratories, California, USA) were used in accordance with the manufacturer’s instructions. Finally, 30-diaminobenzidine tetrahydrochloride (DAB kit, Roche, Basel, CH) was used to visualise peroxidase activity and the sections counterstained with haematoxylin, dehydrated, mounted in DPX and imaged by light microscopy. An isotype matched IgG antibody was used as a negative control.

*Statistical analysis*

All data presented are from at least 3 independent experiments unless stated otherwise and results expressed as mean ± standard deviation (SD). Pairwise differences were measured using Student’s *t*-test whilst group wise comparisons were calculated using one-way ANOVA with appropriate post-hoc test using Graphpad Prism v9.3 (GraphPad, La Jolla, CA) and statistical significance was assumed if p<0.05.

*References*

1. Dickson MA, Hahn WC, Ino Y, et al. Human keratinocytes that express hTERT and also bypass a p16(INK4a)-enforced mechanism that limits life span become immortal yet retain normal growth and differentiation characteristics. *Mol Cell Biol.* 2000;20(4):1436-1447.

2. Harding AL, Murdoch C, Danby S, et al. Determination of Chemical Irritation Potential Using a Defined Gene Signature Set on Tissue-Engineered Human Skin Equivalents. *JID Innov.* 2021;1(2):100011.
