# Supplementary material for: c‐Src activation as a potential marker of chemical‐induced skin irritation using tissue‐engineered skin equivalents
Source: Exp Dermatol. 2022 Dec 11;32(2):220–5. doi: 10.1111/exd.14719 (PMC10946902; doi:10.1111/exd.14719)
Supplement: Supplementary file 2 — Figure S1. Chemical skin irritants induce phosphorylation of p65 NFκB. Figure S2. Phosphokinase array map showing target/control coordinates and antibody‐specific phosphorylation sites. [file EXD-32-220-s002.docx]

**c-Src activation as a potential marker of chemical-induced skin irritation using tissue-engineered skin equivalents**

Amy L. Harding^1^, H E. Colley^1*^, Inmaculada Barragan Vazquez^1^, Simon Danby^2^, Md Zober Hasan^3^, Hirofumi Nakanishi^3^, Tetsuo Furuno^3^ and Craig Murdoch^1^

**Supplemental Figures**

CON

LA

MP

Co-DEA

CIN

CAP


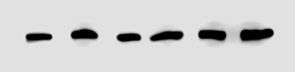

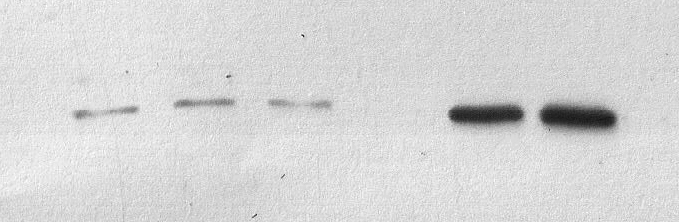


p-p65

Total

p65

**A**

**B**

**Supplementary Figure 1. Chemical skin irritants induce phosphorylation of p65 NFκB.** Immuno-blot analysis of HSE for phospho-p65 abundance compared to total p65 upon stimulation with water control (con), LA, MP, Co-DEA, CIN and CAP for 15 minutes (A). Densitometry analysis showing fold change in phospho-p65 relative to total p65 (B).


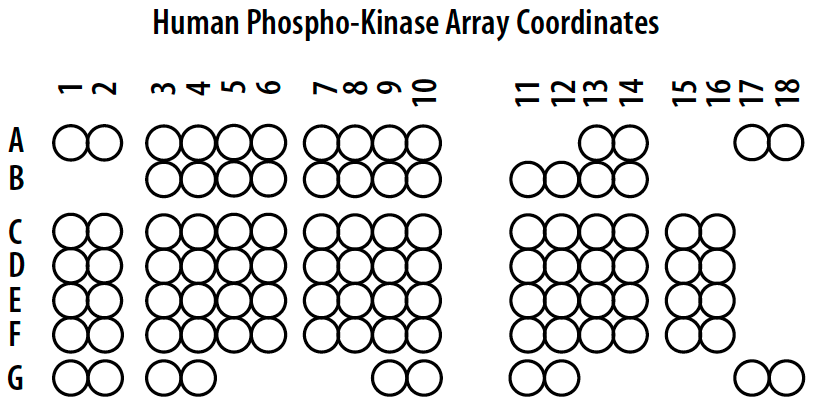


**Supplementary Figure 2.** Phosphokinase array map showing target/control coordinates and antibody-specific phosphorylation sites.
